# The reach of the genome signature in prokaryotes

**DOI:** 10.1186/1471-2148-6-84

**Published:** 2006-10-13

**Authors:** Mark WJ van Passel, Eiko E Kuramae, Angela CM Luyf, Aldert Bart, Teun Boekhout

**Affiliations:** 1 Centraalbureau voor Schimmelcultures (CBS), Uppsalalaan 8, Utrecht, The Netherlands; 2 Bioinformatics Laboratory, Academic Medical Center, University of Amsterdam, the Netherlands; 3 Center for Infection and Immunity Amsterdam (CINIMA), Department of Medical Microbiology, Academic Medical Center, University of Amsterdam, the Netherlands; 4 Department of Biochemistry and Molecular Biophysics, University of Arizona, POBox 210088, Tucson, Arizona, USA

## Abstract

**Background:**

With the increased availability of sequenced genomes there have been several initiatives to infer evolutionary relationships by whole genome characteristics. One of these studies suggested good congruence between genome synteny, shared gene content, 16S ribosomal DNA identity, codon usage and the genome signature in prokaryotes. Here we rigorously test the phylogenetic signal of the genome signature, which consists of the genome-specific relative frequencies of dinucleotides, on 334 sequenced prokaryotic genome sequences.

**Results:**

Intrageneric comparisons show that in general the genomic dissimilarity scores are higher than in intraspecific comparisons, in accordance with the suggested phylogenetic signal of the genome signature. Exceptions to this trend, (*Bartonella *spp., *Bordetella *spp., *Salmonella *spp. and *Yersinia *spp.), which have low average intrageneric genomic dissimilarity scores, suggest that members of these genera might be considered the same species. On the other hand, high genomic dissimilarity values for intraspecific analyses suggest that in some cases (e.g.*Prochlorococcus marinus*, *Pseudomonas fluorescens, Buchnera aphidicola *and *Rhodopseudomonas palustris*) different strains from the same species may actually represent different species. Comparing 16S rDNA identity with genomic dissimilarity values corroborates the previously suggested trend in phylogenetic signal, albeit that the dissimilarity values only provide low resolution.

**Conclusion:**

The genome signature has a distinct phylogenetic signal, independent of individual genetic marker genes. A reliable phylogenetic clustering cannot be based on dissimilarity values alone, as bootstrapping is not possible for this parameter. It can however be used to support or refute a given phylogeny and resulting taxonomy.

## Background

With the availability of an ever-increasing number of whole genome sequences, evolutionary history can be analysed genetically on a more comprehensive level. Among microorganisms, phylogenetic relationships have traditionally been defined by phenotypic characters. With current comparative genomics, evolutionary distances may be inferred more thoroughly, independent of variable expression profiles or morphological characteristics. Recently, a number of studies suggested taxonomy should be based on whole genomic DNA content in order to more properly reflect evolutionary relationships [1-4]. These studies concur with previous observations by Coenye and Vandamme, who tested phylogenetic consistency of a number of parameters in the lactic acid bacteria, including 16S rDNA identity, genome synteny, identity of several house-keeping genes, codon usage and the genome signature similarity. They concluded that these genomic parameters largely agree with each other [5].

The latter parameter, the genome signature, is a compositional parameter reflecting the dinucleotide relative abundance values, which are similar between closely related species, and dissimilar between non-related species [6-8]. The difference in genome signature between two sequences is expressed by the genomic dissimilarity (δ*), which is the average absolute dinucleotide relative abundance difference between two sequences. This parameter is suitable for intragenomic detection of putative horizontally acquired sequences [7,9], and recently an online tool was made available to detect compositional dissimilarity between input sequences and representative whole genome sequences [10], which has shown for example that plasmids are more dissimilar in composition compared to their host genomes than previously anticipated [11], and that genomic islands can be clustered by similarities in dinucleotide composition [12]. Although genome signature analyses have been used for both intragenomic comparisons [7,10-12] and for intergenomic analyses [5,8,13,14], these analyses were predominantly performed on relatively few, or only partial, prokaryotic genomes. While genome signatures had been analysed prior to the genomic era [6], intra- and intergenomic comparisons based on the genome signature between eukaryotes have been scarce [15]. However, a recent study on several eukaryotic genome sequences (i.e. from human, mouse, rat, fruit fly and nematode) based on short range correlations in DNA sequences, showed that in most cases, chromosomes originating from the same species cluster together [16].

In order to assess the applicability of the genome signature in phylogenetic analyses in general, we performed a comprehensive interchromosomal comparison of prokaryotic genome signatures. Using 334 prokaryotic genome sequences, we tested the congruence of the genome signature with the current genus and species nomenclature, and compared the signal of the genome signature to the phylogenetic signal of 16S rDNA identity scores.

## Results

### Prokaryotic whole genome signature comparisons

We calculated the genomic dissimilarity values (δ*) between all 334 available prokaryotic genome sequences available at the time of analysis (May, 2006). We partitioned this large dataset to three smaller subsets to investigate specific topics (see below, see Additional Files 1, 2, 3, 4): the δ* values between chromosomes from the same genus (containing 36 bacterial and 4 archaeal genera: 130 different species in total), the δ* values between chromosomes from the same species (containing 33 bacterial species: 111 different strains in total), and δ* values of prokaryotes with multiple chromosomes (7 different genera, 16 different species). Genera are defined as organisms with the same genus name, and species are defined as organisms with the same genus name and the same specific designation, with two exceptions: *Shigella *spp., which are considered the same species as *Escherichia coli *[17], and the *Bacillus cereus *cluster, consisting of *Bacillus anthracis*, *Bacillus cereus *and *Bacillus thuringensis*, which are also considered the same species [18,19]. Indeed, for both these exceptions, intraclade δ* values are very low as mentioned previously [11,13].

### Intrageneric compositional comparison of whole genomes

**Figure 1:**
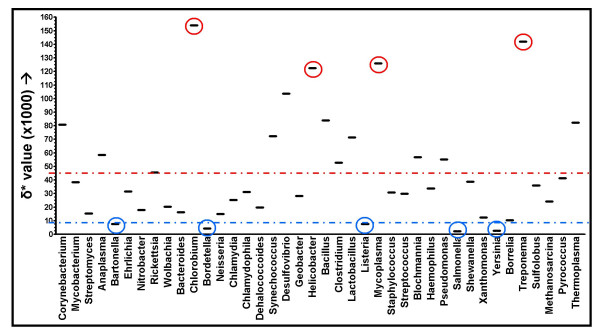
Intrageneric comparison of the genome signature within 40 prokaryotic genera. Species with high genomic dissimilarity scores are in red circles (11: *Chlorobium*, 20: *Helicobacter*, 25; *Mycoplasma *and 36: *Treponema*) species with low genomic dissimilarity scores are in blue circles (5: *Bartonella*, 12: *Bordetella*, 31: *Salmonella *and 34: *Yersinia*). The average genomic dissimilarity between different species of the same genus is 0.046 (red line). The blue line represents the average intraspecific genomic dissimilarity from figure 2 (δ* = 0.009).

Average intrageneric δ* values for 40 different genera consisting of both Archaea and Bacteria (Additional File 1) are depicted in Figure 1, indicating a large variation in the genome signature, with an average intrageneric δ* score of 0.046. All chromosomes from different species of the same genus were compared for these analyses, but intraspecific comparisons were carried out separately. Extreme δ* values of intergeneric comparisons were highlighted in red (high δ*) and blue (low δ*).

The four genera with very high intrageneric δ* values are *Chlorobium*, *Helicobacter*, *Mycoplasma *and *Treponema*. Although the *Chlorobium *genome sequences are available, the sequences are unpublished, and it is therefore unclear why the genome signature between species of the same genus would differ so greatly. From the general genomic attributes at NCBI [20], it is obvious however that the genomes of *Chlorobium chlorochromatii *and *Chlorobium tepidum *differ substantially in GC% content (44.3% and 56%, respectively), and to a lesser extent in genome size (2.57 Mbp and 2.15 Mbp, respectively), suggesting a substantial phylogenetic distance. This however conflicts with the 16S rDNA sequence identity of 92%.

The reason for the high genomic dissimilarities between the genomes of *Helicobacter hepaticus *and the two *Helicobacter pylori *strains are possibly due to substantial differences in gene complement (possibly due to horizontal gene transfer) between members of this genus [21]. A large phylogenetic distance between these species has been observed previously [22,23], and whole genome analyses by Dewhirst and co-workers support a phylogeny that places *H. hepaticus *more closely related to *Wolinella succinogenes *than to *Helicobacter pylori *[24]. The latter study actually supports HGT of 16S sequences, thus obscuring single locus-based phylogenies for this genus.

The *Mycoplasma *spp. (including *Ureaplasma parvum*) and the *Treponema *spp. show low intrageneric 16S rDNA sequence identity scores (between 70–98.3% sequence identity and 89.1% identity, respectively), which had already been noted for a subset of the *Mycoplasma spp*. genome sequences [8,13]. These two genera also show large intrageneric phylogenetic distances in a Tree of Life study based on universally conserved genes [22].

The four genera with very low intrageneric δ* values are *Bartonella *(0.0077), *Bordetella *(0.0042), *Salmonella *(0.0023) and *Yersinia *(0.0025). These values are lower than the average intraspecific δ* values (see below). All species that constitute these four genera have nearly identical 16S rDNA sequences (i.e. show 98.9%, 99.8%, 98.2% and 99.9% intrageneric identity, respectively), thus corroborating the established close, possibly intraspecific, evolutionary relationship.

### Intraspecific compositional comparisons of whole genomes

**Figure 2:**
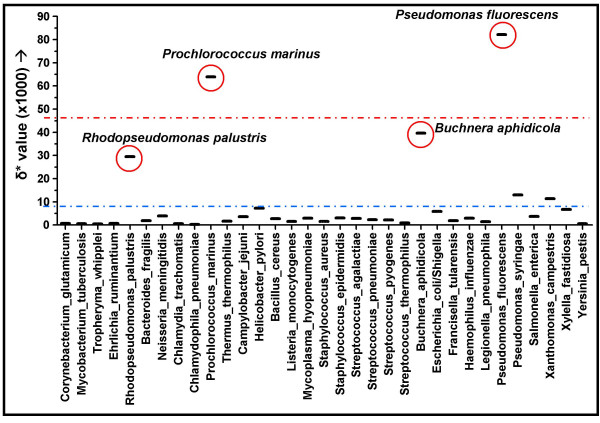
Intraspecific comparisons of genome signature of 33 bacterial species. Species with high genomic dissimilarity scores are in red circles. The average genomic dissimilarity between genomes of the same species is 0.009 (blue line). The red line depicts the average intrageneric genomic dissimilarity from Figure 1 (δ* = 0.046).

Although no archaeal genome sequences of the same species are currently available, for 33 bacterial species there are two or more genome sequences available (Additional File 2), allowing the detection of variation of δ* of genome sequences from the same species. The average intraspecific genomic dissimilarity values are depicted in Figure 2, with an average intraspecific δ* of 0.009. Four clear outliers are detected with substantially higher δ* values (red circles in Figure 2), originating from intraspecific comparisons between the genomes from *Rhodopseudomonas palustris*, *Prochlorococcus marinus*, *Buchnera aphidicola *and *Pseudomonas fluorescens *(intraspecific δ* values of 0.0295, 0.0639, 0.0396 and 0.0822, respectively).

Except for *B. aphidicola*, for which the intraspecific 16S rDNA sequence identity is low for members of the same species (91%), the other 16S rDNA sequence identities are >96%. However, for the *P. marinus *genomes, the range of genomic GC percentages is considerable (30.8–50.7% GC), and the genome sizes differ substantially (1.66–2.41 Mbp), suggesting that these organisms may not be the same species, they contain similar 16S rDNA sequences due to HGT of these loci or that acquisition of large amounts of anomalous DNA plays an important role in the genome organization and nucleotide composition.

For both *P. fluorescens *genomes, a difference in genome size is observed (>600 Mbp), but the difference in GC percentage is relatively small.

### Congruence between genomic dissimilarity values and 16S rDNA identity values of different bacterial species

**Figure 3:**
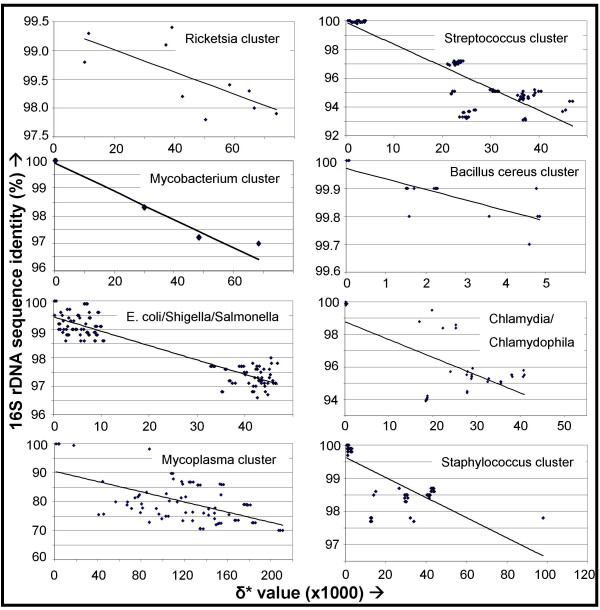
Congruence between 16S rDNA sequence identity (y-axis) and δ* (x-axis) within different groups (note the different scales on the axes). See Additional File 3 for more information.

It had been suggested previously that various genomic parameters are congruent in their phylogenetic signal [5]. We compared eight sets of genomic dissimilarity values with 16S rDNA sequence identity scores between eight different groups of species; one group containing the *B. cereus *cluster, and seven groups of several related species (the *Chlamydia*/*Chlamydophila *clade, the genus *Mycoplasma*, the genus *Staphylococcus*, the *E. coli*/*Shigella*/*Salmonella *clade, the genus *Mycobacterium*, the genus *Ricketsia*, the genus *Lactobacillus *and the genus *Streptococcus*; seven groups in total. see also Additional File 3). An inverse correlation between these two parameters is observed (Fig. 3, note the differences in scale of the 16S rDNA identity and δ*), although incongruities are visible (e.g. within the *E. coli*/*Shigella*/*Salmonella *clade).

### Whole genome comparisons of prokaryotic chromosomes of species with multiple chromosomes

**Figure 4:**
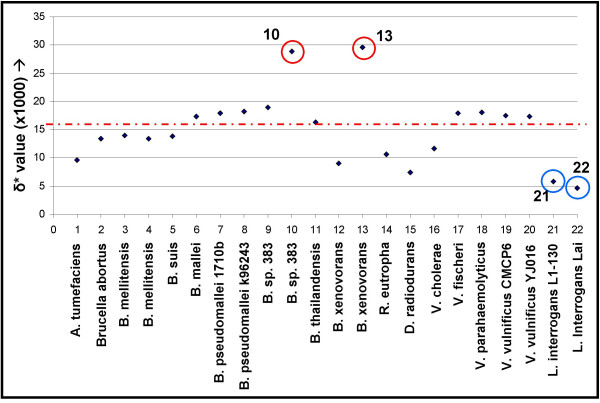
Intragenomic comparisons of the genome signature of 20 bacterial species (see Additional File 4). Highlighted are 10 (*Burkholderia *sp. 383 chromosome 1 vs. 3), 13 (*Burkholderia xenovorans *LB400 chromosome 1 vs. 3), 21 (*Leptospira interrogans *serovar Copenhageni str. Fiocruz L1–130 chromosome I vs. II) and 22 (*Leptospira interrogans *serovar Lai str. 56601 chromosome I vs. II). The average genomic dissimilarity between chromosomes of the same species is 0.016 (red line).

Sixteen prokaryotic species contain more than one chromosome and the δ* values between the two chromosomes of the same strains are illustrated in Figure 4 (for the species list, see Additional File 4). On average, the δ* value between two chromosomes from the same species is 0.0156. Four outliers are circled; the two higher outliers are comparisons between chromosomes 1 and 3 from *Burkholderia *spp. 383 and chromosomes 1 and 3 from *Burkholderia xenovorans*, indicating a very high genomic dissimilarity between these chromosomes and thereby suggesting an exogenous origin for chromosome 3 in these species (δ* is 0.0289 and 0.0296 for *B*. spp. 383 and *B. xenovorans*, respectively). The two lower outliers are the two different chromosome I and chromosome II comparisons from the two *Leptospira interrogans *species. The intragenomic δ* value is 0.0059 for *L. interrogans *serovar Copenhageni and 0.0047 for *L. interrogans *serovar Lai suggesting a longer shared evolutionary history.

## Conclusion

Traditionally, the assignment of taxonomical appropriate names to microbes was based on phenotypic characters, such as Gram stains or the possession of cell walls. Currently, with over 350 whole genomes sequenced, there is ongoing debate to re-evaluate the prokaryotic species definition [2,25]. One such attempt, considering different genomic parameters, was performed by Coenye and Vandamme [5], who compared different phylogenetic approaches on the lactic acid bacteria. They concluded that the different approaches agreed well, although these do not necessarily provide much additional information about phylogenetic relationships. In our study, we analysed the overall consistency of the phylogenetic signal of the genome signature in 334 prokaryotic. We tested the congruence between the δ* intragenerically, intraspecifically, and the δ* values with their corresponding 16S rDNA sequence identity values.

For some species (*E. coli*/*Shigella *group, *Bacillus cereus *cluster) it was known that they are probably the same species, and the low values for δ* corroborate this [11,13]. Comparison of the intrageneric δ* (Fig. 1) and intraspecific δ* (Fig. 2) shows various intrageneric values to be well in the range of intraspecific values, suggesting that there are more clusters that may actually constitute one species. This is the case for the different *Bartonella *spp., *Bordetella *spp., *Salmonella *spp. and *Yersinia *spp., This may also hold true for *M. bovis *and *M. tuberculosis*, and the *L. innocua *and *L. monocytogenes *species. For these six groups, the 16S rDNA data support the notion of very close phylogenetic relationships.

In contrast, four extreme intraspecific δ* values are within the intrageneric range. The different *B. aphidicola *species display high genomic dissimilarity values as well as low 16S rDNA sequence identity scores, suggesting these might actually be different species. This is in agreement with an estimated divergence time of over 150 million years [26]. The reason why the different species of *R. palustris*, *P. marinus *and *P. fluorescens *display high δ* values, while the ribosomal sequences of the individual species are nearly identical remains unclear, although very long branches have been observed between members of these species in different phylogenetic studies [22,23]. It is of note that between the different *P. marinus *genomes substantial differences in size and GC percentage are observed.

Generally, an inverse relation between δ* and 16S is found, but the perceived resolution of this relation seems low and therefore δ* values alone seem insufficient to infer reliable phylogenetic relationships. Also, it is not possible to infer a reliable phylogenetic clustering based on distance matrices as bootstrapping is not possible.

For some prokaryotic species containing multiple chromosomes it had been suggested that the secondary chromosome may have been acquired via horizontal gene transfer [12,27]. We find that the genomic dissimilarity between the two primary chromosomes in bacteria is generally low, but it is higher than the genomic dissimilarity between chromosomes from the same species, supporting the HGT hypothesis. Intragenomic dispersal of DNA can ameliorate the dissimilarity in genome signature, obscuring compositional dissimilarities over time [12]. A consequence of this is that different chromosomes found in metagenomic analyses can not readily be grouped into genomes for prokaryotes, though this is a minor problem as most prokaryotes have single chromosome genomes. We find that each intragenomic comparison of the two chromosomes of the different *Vibrio *genomes yields a higher δ* value than the average of δ* = 0.009 in intraspecific comparisons (data not shown). Secondary chromosomes are present in all sequenced genomes of *Vibrio *spp., and if they have been present in each genome since the split of the different *Vibrio *spp. the different chromosomes in each genome should have had ample time to ameliorate towards more similar dinucleotide frequencies. The fact that the different chromosomes of each *Vibrio *genome are still dissimilar from each other in composition may be caused by an instable chromosome II, which is known to be less well-conserved between the different *Vibrio *species than chromosome I [28].

The precise origin of the genome signature is still unknown. For the GC percentage it has been suggested that certain environmental conditions shape the nucleotide composition [29]. This has also been found to be the case for the genome signature [30], although the exact effect of different conditions on different genome sequences remains unknown. It is likely that mutational pressures direct the shape of the genome signature, but the fact that secondary chromosomes in most cases remain dissimilar from the primary chromosomes underscores our lack of understanding of the factors that shape the nucleotide composition.

In conclusion, the genome signature is more similar between closely related species, and increases with larger phylogenetic distances, but this relation seems inadequate to infer phylogenetic relationships by itself. Unfortunately, distance matrices based on single values, as is the case with δ* scores, are not amenable to bootstrapping, so robust phylogenetic analysis can not be inferred from δ* values for prokaryotes. This parameter does however have a strong phylogenetic signal and can therefore be used to support or contradict a given phylogeny and resulting taxonomy. The combination of δ* and 16S rDNA data given above for *Mycobacteria*, *Listeria, Prochlorococcus *and *Buchnera *provide convincing evidence for a re-evaluation of these taxonomic relationships. Also, if there are no additional ways to infer relationships (e.g. in the absence of comparable markers, as with multiple chromosomes in metagenomic analyses) the genome signature may help to cluster chromosomes, although the intragenomic δ* scores can be relatively high in multichromosomal genomes from prokaryotes.

## Methods

### Sequences and data analyses

Prokaryotic chromosomal sequences were obtained from NCBI [20] (Additional Files 1 to 4, August 2005). Genome signature comparisons were performed using the online tool Compare_Islands [10,12,22], and all prokaryote genome comparisons are available at the Compare_Islands website [31]. The 16S rDNA sequences were obtained from the NCBI website [20] and trimmed after alignment by using the MEGA3 software package [32]. Identity percentages were calculated using MATGAT with the BLOSUM50 scoring matrix [33].

## Authors' contributions

MWJvP, EEK and TB conceived the study and carried out the analyses. ACML carried out the bioinformatical analyses. MWJvP, EEK, AB and TB analysed the data and wrote the manuscript. All authors have read and approved the final manuscript.

## Supplementary Material

Additional File 1 Prokaryotic intrageneric genome signature comparisons. Click here for file

Additional File 2 Prokaryotic intraspecific genome signature comparisons (the δ* scores are available from the large matrix at ). Click here for file

Additional File 3 Prokaryotic intrageneric genome signature comparisons. In these matrices; the 16S rDNA sequence identity (in %) is given in the top-right half, and the δ* (×1000) are given in the lower-left part. Click here for file

Additional File 4 Prokaryotic intragenomic genome signature comparisons for species that carry more than 1 chromosome. The numbers correspond to the comparisons depicted in Figure 4. Click here for file
