# Supplementary material for: The reach of the genome signature in prokaryotes
Source: BMC Evol Biol. 2006 Oct 13;6:84. doi: 10.1186/1471-2148-6-84 (PMC1621082; doi:10.1186/1471-2148-6-84)
Supplement: Additional File 1 — Prokaryotic intrageneric genome signature comparisons. [file 1471-2148-6-84-S1.pdf]

Additional File 1

Prokaryotic intrageneric genome signature comparisons. Note that some genera consist of only one species-type (e.g. Tropheryma). These are not considered in the intrageneric comparisons, but instead in the intraspecific comparisons.

		Numbers corresponding to Fig. 1		Accession numbers	Size (bp)
**Bacteria**					
	**Actinobacteria**				
		**1**	Corynebacterium_diphtheriae_NCTC_13129	NC_002935	2488635
			Corynebacterium_efficiens_YS-314	NC_004369	3147090
			Corynebacterium_glutamicum_ATCC_13032	NC_003450	3309401
			Corynebacterium_glutamicum_ATCC_13032	NC_006958	3282708
			Corynebacterium_jeikeium_K411	NC_007164	2462499
		**2**	Mycobacterium_avium_subsp_paratuberculosis_str_k10	NC_002944	4829781
			Mycobacterium_bovis_AF2122/97	NC_002945	4345492
			Mycobacterium_leprae_TN	NC_002677	3268203
			Mycobacterium_tuberculosis_CDC1551	NC_002755	4403837
			Mycobacterium_tuberculosis_H37Rv	NC_000962	4411532
		**3**	Streptomyces_avermitilis_MA-4680	NC_003155	9025608
			Streptomyces_coelicolor_A3(2)	NC_003888	8667507
		*****	Tropheryma_whipplei_TW08/27	NC_004551	925938
			Tropheryma_whipplei_str_Twist	NC_004572	927303
	**Alphaproteobacteria**				
		**4**	Anaplasma_marginale_str_St_Maries	NC_004842	1197687
			Anaplasma_phagocytophilum_HZ	NC_007797	1471282
		**5**	Bartonella_henselae_str_Houston-1	NC_005956	1931047

		Bartonella_quintana_str_Toulouse	NC_005955	1581384
	**6**	Ehrlichia_canis_str_Jake	NC_007354	1315030
		Ehrlichia_chaffeensis_str_Arkansas	NC_007799	1176248
		Ehrlichia_ruminantium_str_Gardel	NC_006831	1499920
		Ehrlichia_ruminantium_str_Welgevonden	NC_005295	1516355
		Ehrlichia_ruminantium_str_Welgevonden	NC_006832	1512977
	**7**	Nitrobacter_hamburgensis_X14	NC_007964	4406967
		Nitrobacter_winogradskyi_Nb-255	NC_007406	3402093
	*****	Rhodopseudomonas_palustris_BisB18	NC_007925	5513844
		Rhodopseudomonas_palustris_BisB5	NC_007958	4892717
		Rhodopseudomonas_palustris_CGA009	NC_005296	5459213
		Rhodopseudomonas_palustris_HaA2	NC_007778	5331656
	**8**	Rickettsia_bellii_RML369-C	NC_007940	1522076
		Rickettsia_conorii_str_Malish_7	NC_003103	1268755
		Rickettsia_felis_URRWXCal2	NC_007109	1485148
		Rickettsia_prowazekii_str_Madrid_E	NC_000963	1111523
		Rickettsia_typhi_str_Wilmington	NC_006142	1111496
	**9**	Wolbachia_endosymbiont_of_Drosophila_melanogaster	NC_002978	1267782
		Wolbachia_endosymbiont_strain_TRS_of_Brugia_malayi	NC_006833	1080084
**Bacteroidetes/Chlorobi**				
	**10**	Bacteroides_fragilis_NCTC_9343	NC_003228	5205140
		Bacteroides_fragilis_YCH46	NC_006347	5277274
		Bacteroides_thetaiotaomicron_VPI-5482	NC_004663	6260361
	**11**	Chlorobium_chlorochromatii_CaD3	NC_007514	2572079
		Chlorobium_tepidum_TLS	NC_002932	2154946

**Betaproteobacteria**				
	**12**	Bordetella_bronchiseptica_RB50	NC_002927	5339179
		Bordetella_parapertussis_12822	NC_002928	4773551
		Bordetella_pertussis_Tohama_I	NC_002929	4086189
	**13**	Neisseria_gonorrhoeae_FA_1090	NC_002946	2153922
		Neisseria_meningitidis_MC58	NC_003112	2272360
		Neisseria_meningitidis_Z2491	NC_003116	2184406
**Chlamydiae/Verrucomicrobia**				
	**14**	Chlamydia_muridarum_Nigg	NC_002620	1072950
		Chlamydia_trachomatis_A/HAR-13	NC_007429	1044459
		Chlamydia_trachomatis_D/UW-3/CX	NC_000117	1042519
	**15**	Chlamydophila_abortus_S26/3	NC_004552	1144377
		Chlamydophila_caviae_GPIC	NC_003361	1173390
		Chlamydophila_felis_Fe/C-56	NC_007899	1166239
		Chlamydophila_pneumoniae_AR39	NC_002179	1229853
		Chlamydophila_pneumoniae_CWL029	NC_000922	1230230
		Chlamydophila_pneumoniae_J138	NC_002491	1226565
		Chlamydophila_pneumoniae_TW-183	NC_005043	1225935
**Chloroflexi**				
	**16**	Dehalococcoides_ethenogenes_195	NC_002936	1469720
		Dehalococcoides_sp_CBDB1	NC_007356	1395502
**Cyanobacteria**				
	*****	Prochlorococcus_marinus_str_MIT_9312	NC_007577	1709204
		Prochlorococcus_marinus_str_MIT_9313	NC_005071	2410873
		Prochlorococcus_marinus_str_NATL2A	NC_007335	1842899
		Prochlorococcus_marinus_subsp_marinus_str_CCMP1375	NC_005042	1751080
		Prochlorococcus_marinus_subsp_pastoris_str_CCMP1986	NC_005072	1657990

	**17**	Synechococcus_elongatus_PCC_6301	NC_006576	2696255
		Synechococcus_elongatus_PCC_7942	NC_007604	2695903
		Synechococcus_sp_CC9605	NC_007516	2510659
		Synechococcus_sp_CC9902	NC_007513	2234828
		Synechococcus_sp_WH_8102	NC_005070	2434428
**Deinococcus-Thermus**				
	*****	Thermus_thermophilus_HB27	NC_005835	1894877
		Thermus_thermophilus_HB8	NC_006461	1849742
**Deltaproteobacteria**				
	**18**	Desulfovibrio_desulfuricans_G20	NC_007519	3730232
		Desulfovibrio_vulgaris_subsp_vulgaris_str_Hildenborough	NC_002937	3570858
	**19**	Geobacter_metallireducens_GS-15	NC_007517	3997420
		Geobacter_sulfurreducens_PCA	NC_002939	3814139
**Epsilonproteobacteria**				
	*****	Campylobacter_jejuni_RM1221	NC_003912	1777831
		Campylobacter_jejuni_subsp_jejuni_NCTC_11168	NC_002163	1641481
	**20**	Helicobacter_hepaticus_ATCC_51449	NC_004917	1799146
		Helicobacter_pylori_26695	NC_000915	1667867
		Helicobacter_pylori_J99	NC_000921	1643831
**Firmicutes**				
	**21**	Bacillus_anthracis_A2012,_unfinished_sequence	NC_003995	5051677
		Bacillus_anthracis_str_Ames	NC_003997	5227293
		Bacillus_anthracis_str_Ames_AncestorcleanNC_007404fna	NC_007530	5227419
		Bacillus_anthracis_str_Sterne	NC_005945	5228663
		Bacillus_cereus_ATCC_10987	NC_003909	5224283
		Bacillus_cereus_ATCC_14579	NC_004722	5411809
		Bacillus_cereus_E33L	NC_006274	5300915

	Bacillus_clausii_KSM-K16	NC_006582	4303871
	Bacillus_halodurans_C-125	NC_002570	4202352
	Bacillus_licheniformis_ATCC_14580	NC_006270	4222334
	Bacillus_licheniformis_ATCC_14580	NC_006322	4222645
	Bacillus_subtilis_subsp_subtilis_str_168	NC_000964	4214630
	Bacillus_thuringiensis_serovar_konkukian_str_97-27	NC_005957	5237682
**22**	Clostridium_acetobutylicum_ATCC_824	NC_003030	3940880
	Clostridium_perfringens_str_13	NC_003366	3031430
	Clostridium_tetani_E88	NC_004557	2799251
**23**	Lactobacillus_acidophilus_NCFM	NC_006814	1993564
	Lactobacillus_johnsonii_NCC_533	NC_005362	1992676
	Lactobacillus_plantarum_WCFS1	NC_004567	3308274
	Lactobacillus_sakei_subsp_sakei_23K	NC_007576	1884661
	Lactobacillus_salivarius_subsp_salivarius_UCC118	NC_007929	1827111
**24**	Listeria_innocua_Clip11262	NC_003212	3011208
	Listeria_monocytogenes_EGD-e	NC_003210	2944528
	Listeria_monocytogenes_str_4b_F2365	NC_002973	2905187
**25**	Mycoplasma_capricolum_subsp_capricolum_ATCC_27343	NC_007633	1010023
	Mycoplasma_gallisepticum_R	NC_004829	996422
	Mycoplasma_genitalium_G-37	NC_000908	580074
	Mycoplasma_hyopneumoniae_232	NC_006360	892758
	Mycoplasma_hyopneumoniae_7448	NC_007332	920079
	Mycoplasma_hyopneumoniae_J	NC_007295	897405
	Mycoplasma_mobile_163K	NC_006908	777079
	Mycoplasma_mycoides_subsp_mycoides_SC_str_PG1	NC_005364	1211703
	Mycoplasma_penetrans_HF-2	NC_004432	1358633
	Mycoplasma_pneumoniae_M129	NC_000912	816394
	Mycoplasma_pulmonis_UAB_CTIP	NC_002771	963879
	Mycoplasma_synoviae_53	NC_007294	799476

		Ureaplasma_parvum_serovar_3_str_ATCC_700970	NC_002162	751719
	26	Staphylococcus_aureus_RF122	NC_007622	2742531
		Staphylococcus_aureus_subsp_aureus_COL	NC_002951	2809422
		Staphylococcus_aureus_subsp_aureus_MRSA252	NC_002952	2902619
		Staphylococcus_aureus_subsp_aureus_MSSA476	NC_002953	2799802
		Staphylococcus_aureus_subsp_aureus_MW2	NC_003923	2820462
		Staphylococcus_aureus_subsp_aureus_Mu50	NC_002758	2878529
		Staphylococcus_aureus_subsp_aureus_N315	NC_002745	2814816
		Staphylococcus_aureus_subsp_aureus_NCTC_8325	NC_007795	2821361
		Staphylococcus_aureus_subsp_aureus_USA300	NC_007793	2872769
		Staphylococcus_epidermidis_ATCC_12228	NC_004461	2499279
		Staphylococcus_epidermidis_RP62A	NC_002976	2616530
		Staphylococcus_haemolyticus_JCSC1435	NC_007168	2685015
		Staphylococcus_saprophyticus_subsp_saprophyticus	NC_007350	2516575
	27	Streptococcus_agalactiae_2603V/R	NC_004116	2160267
		Streptococcus_agalactiae_A909	NC_007432	2127839
		Streptococcus_agalactiae_NEM316	NC_004368	2211485
		Streptococcus_mutans_UA159	NC_004350	2030921
		Streptococcus_pneumoniae_R6	NC_003098	2038615
		Streptococcus_pneumoniae_TIGR4	NC_003028	2160837
		Streptococcus_pyogenes_M1_GAS	NC_002737	1852441
		Streptococcus_pyogenes_MGAS10394	NC_006086	1899877
		Streptococcus_pyogenes_MGAS315	NC_004070	1900521
		Streptococcus_pyogenes_MGAS5005	NC_007297	1838554
		Streptococcus_pyogenes_MGAS6180	NC_007296	1897573
		Streptococcus_pyogenes_MGAS8232	NC_003485	1895017
		Streptococcus_pyogenes_SSI-1	NC_004606	1894275
		Streptococcus_thermophilus_CNRZ1066	NC_006449	1796226
		Streptococcus_thermophilus_LMG_18311	NC_006448	1796846
**Gammaproteobacteria**				

*	Buchnera_aphidicola_str_APS_(Acyrthosiphon_pisum)	NC_002528	640681
	Buchnera_aphidicola_str_Bp_(Baizongia_pistaciae)	NC_004545	615980
	Buchnera_aphidicola_str_Sg_(Schizaphis_graminum)	NC_004061	641454
**28**	Candidatus_Blochmannia_floridanus	NC_005061	705557
	Candidatus_Blochmannia_pennsylvanicus_str_BPEN	NC_007292	791654
*	Escherichia_coli_CFT073	NC_004431	5231428
	Escherichia_coli_K12	NC_000913	4639675
	Escherichia_coli_O157:H7	NC_002695	5498450
	Escherichia_coli_O157:H7_EDL933	NC_002655	5528445
	Escherichia_coli_UTI89	NC_007946	5065741
	Escherichia_coli_W3110_DNA	AC_000091	4646332
	Shigella_boydii_Sb227	NC_007613	4519823
	Shigella_dysenteriae_Sd197	NC_007606	4369232
	Shigella_flexneri_2a_str_2457T	NC_004741	4599354
	Shigella_flexneri_2a_str_301	NC_004337	4607203
	Shigella_sonnei_Ss046	NC_007384	4825265
*	Francisella_tularensis_subsp_holarctica	NC_007880	1895994
	Francisella_tularensis_subsp_tularensis	NC_006570	1892819
**29**	Haemophilus_ducreyi_35000HP	NC_002940	1698955
	Haemophilus_influenzae_86-028NP	NC_007146	1913428
	Haemophilus_influenzae_Rd_KW20	NC_000907	1830138
*	Legionella_pneumophila_str_Lens	NC_006369	3345687
	Legionella_pneumophila_str_Paris	NC_006368	3503610
	Legionella_pneumophila_subsp_pneumophila_str_Philadelphia_1	NC_002942	3397754
**30**	Pseudomonas_aeruginosa_PAO1	NC_002516	6264403
	Pseudomonas_fluorescens_Pf-5	NC_004129	7074893
	Pseudomonas_fluorescens_PfO-1	NC_007492	6438405

		Pseudomonas_putida_KT2440	NC_002947	6181863
		Pseudomonas_syringae_pv_phaseolicola_1448A	NC_005773	5928787
		Pseudomonas_syringae_pv_syringae_B728a	NC_007005	6093698
		Pseudomonas_syringae_pv_tomato_str_DC3000	NC_004578	6397126
	**31**	Salmonella_enterica_subsp_enterica_serovar_Choleraesuis_str_SC-B67	NC_006905	4755700
		Salmonella_enterica_subsp_enterica_serovar_Paratypi_A_str_ATCC_9150	NC_006511	4585229
		Salmonella_enterica_subsp_enterica_serovar_Typhi_Ty2	NC_004631	4791961
		Salmonella_enterica_subsp_enterica_serovar_Typhi_str_CT18	NC_003198	4809037
		Salmonella_typhimurium_LT2	NC_003197	4857432
	**32**	Shewanella_denitrificans_OS217	NC_007954	4545906
		Shewanella_oneidensis_MR-1	NC_004347	4969803
	**33**	Xanthomonas_axonopodis_pv_citri_str_306	NC_003919	5175554
		Xanthomonas_campestris_pv_campestris_str_8004	NC_007086	5148708
		Xanthomonas_campestris_pv_campestris_str_ATCC_33913	NC_003902	5076188
		Xanthomonas_campestris_pv_vesicatoria_str_85-10	NC_007508	5178466
		Xanthomonas_oryzae_pv_oryzae_KACC10331	NC_006834	4941439
	*****	Xylella_fastidiosa_9a5c	NC_002488	2679306
		Xylella_fastidiosa_Temecula1	NC_004556	2519802
	**34**	Yersinia_pestis_CO92	NC_003143	4653728
		Yersinia_pestis_KIM	NC_004088	4600755
		Yersinia_pestis_biovar_Medievalis_str_91001	NC_005810	4595065
		Yersinia_pseudotuberculosis_IP_32953	NC_006155	4744671
**Spirochaetes**				
	**35**	Borrelia_burgdorferi_B31	NC_001318	910724
		Borrelia_garinii_PBi_chromosome_linear	NC_006156	904246
	**36**	Treponema_denticola_ATCC_35405	NC_002967	2843201

			Treponema_pallidum_subsp_pallidum_str_Nichols	NC_000919	1138011
**Archaea**					
	**Crenarchaeota**				
		**37**	Sulfolobus_acidocaldarius_DSM_639	NC_007181	2225959
			Sulfolobus_solfataricus_P2	NC_002754	2992245
			Sulfolobus_tokodaii_str_7	NC_003106	2694756
	**Euryarchaeota**				
		**38**	Methanosarcina_acetivorans_C2A	NC_003552	5751492
			Methanosarcina_barkeri_str_fusaro_chromosome_1	NC_007355	4837408
			Methanosarcina_mazei_Go1	NC_003901	4096345
		**39**	Pyrococcus_abyssi_GE5	NC_000868	1765118
			Pyrococcus_furiosus_DSM_3638	NC_003413	1908256
			Pyrococcus_horikoshii_OT3	NC_000961	1738505
		**40**	Thermoplasma_acidophilum_DSM_1728	NC_002578	1564906
			Thermoplasma_volcanium_GSS1	NC_002689	1584804
			**Total number of genome sequences**	209	

***) Genera with only 1 species are excluded from the intrageneric comparisons (these are included in the intraspecific comparisons)**
