# Supplementary material for: The reach of the genome signature in prokaryotes
Source: BMC Evol Biol. 2006 Oct 13;6:84. doi: 10.1186/1471-2148-6-84 (PMC1621082; doi:10.1186/1471-2148-6-84)
Supplement: Additional File 2 — Prokaryotic intraspecific genome signature comparisons (the δ* scores are available from the large matrix at ). [file 1471-2148-6-84-S2.pdf]

Additional File 1

Prokaryotic intraspecific genome signature comparisons (the δ* scores are available from the large matrix at http://deltarho.amc.nl).

		Number		# of strains	Accession numbers			
**Bacteria**								
	Actinobacteria							
		**1**	Corynebacterium_glutamicum	**2**	NC_003450	NC_006958		
		**2**	Mycobacterium_tuberculosis	**2**	NC_002755	NC_000962		
		**3**	Tropheryma_whipplei	**2**	NC_004551	NC_004572		
	Alphaproteobacteria							
		**4**	Ehrlichia_ruminantium	**3**	NC_006831	NC_005295	NC_006832	
		**5**	Rhodopseudomonas_palustris	**4**	NC_007925	NC_007958	NC_005296	NC_007778
	Bacteroidetes/Chlorobi							
		**6**	Bacteroides_fragilis	**2**	NC_003228	NC_006347		
	Betaproteobacteria							
		**7**	Neisseria_meningitidis	**2**	NC_003112	NC_003116		
	Chlamydiae/ Verrucomicrobia							
		**8**	Chlamydia_trachomatis	**2**	NC_007429	NC_000117		

	**9**	Chlamydophila_pneumoniae	**4**	NC_002179	NC_000922	NC_002491	NC_005043		
Cyanobacteria									
	**10**	Prochlorococcus_marinus	**5**	NC_007577	NC_005071	NC_007335	NC_005042	NC_005072	
Deinococcus-Thermus									
	**11**	Thermus_thermophilus	**2**	NC_005835	NC_006461				
Epsilonproteobacteria									
	**12**	Campylobacter_jejuni	**2**	NC_003912	NC_002163				
	**13**	Helicobacter_pylori	**2**	NC_000915	NC_000921				
Firmicutes									
	**14**	Bacillus_cereus cluster	**8**	NC_003995	NC_003997	NC_007530	NC_005945		
				NC_003909	NC_004722	NC_006274	NC_005957		
	**15**	Listeria_monocytogenes	**2**	NC_003210	NC_002973				
	**16**	Mycoplasma_hyopneumoniae	**3**	NC_006360	NC_007332	NC_007295			
	**17**	Staphylococcus_aureus	**9**	NC_007622	NC_002951	NC_002952	NC_002953	NC_003923	
				NC_002758	NC_002745	NC_007795	NC_007793		
	**18**	Staphylococcus_epidermidis	**2**	NC_004461	NC_002976				
	**19**	Streptococcus_agalactiae	**3**	NC_004116	NC_007432				
	**20**	Streptococcus_pneumoniae	**2**	NC_003098	NC_003028				
	**21**	Streptococcus_pyogenes	**7**	NC_002737	NC_006086	NC_004070	NC_007297	NC_007296	NC_003485
				NC_004606	NC_007296	NC_003485	NC_004606		

	**22**	Streptococcus_thermophilus	**2**	NC_006449	NC_006448				
Gammaproteobacteria									
	**23**	Buchnera_aphidicola	**3**	NC_002528	NC_004545	NC_004061			
	**24**	Escherichia_coli (including Shigellae)	**11**	NC_004431	NC_000913	NC_002695	NC_002655	NC_007946	AC_000091
				NC_007613	NC_007606	NC_004741	NC_004337	NC_007384	
	**25**	Francisella_tularensis	**2**	NC_007880	NC_006570				
	**26**	Haemophilus_influenzae	**2**	NC_007146	NC_000907				
	**27**	Legionella_pneumophila	**3**	NC_006369	NC_006368	NC_002942			
	**28**	Pseudomonas_fluorescens	**2**	NC_004129	NC_007492				
	**29**	Pseudomonas_syringae	**3**	NC_005773	NC_007005	NC_004578			
	**30**	Salmonella_enterica	**4**	NC_006905	NC_006511	NC_004631	NC_003198		
	**31**	Xanthomonas_campestris	**3**	NC_007086	NC_003902	NC_007508			
	**32**	Xylella_fastidiosa	**2**	NC_002488	NC_004556				
	**33**	Yersinia_pestis	**4**	NC_003143	NC_004088	NC_005810			
		**Total number of genome sequences**	**111**						
