# Supplementary material for: The reach of the genome signature in prokaryotes
Source: BMC Evol Biol. 2006 Oct 13;6:84. doi: 10.1186/1471-2148-6-84 (PMC1621082; doi:10.1186/1471-2148-6-84)
Supplement: Additional File 3 — Prokaryotic intrageneric genome signature comparisons. In these matrices; the 16S rDNA sequence identity (in %) is given in the top-right half, and the δ* (×1000) are given in the lower-left part. [file 1471-2148-6-84-S3.pdf]

Additional File 3

Prokaryotic intrageneric genome signature comparisons. In these matrices; the 16S rDNA sequence identity is given in the top-right half, and the δ* (x1000) are given in the lower left part. Note that comparisons are intrageneric, except for the E. coli/Shigella/Salmonella group (which consists of multiple genera) and the Bacillus cereus cluster, which is considered one species.

Prokaryotic species

	1	2	3	4	5
1. Mycobacteriumaviumsubsp.paratuberculosisK-10		98.3	97	98.3	98.3
2. MycobacteriumbovisAF2122/97	30.3		97.2	100	100
3. MycobacteriumlepraeTN	68.3	48.4		97.2	97.2
4. MycobacteriumtuberculosisCDC1551	30.1	0.3	48.6		100
5. MycobacteriumtuberculosisH37Rv	30.1	0.4	48.5	0.5	

	1	2	3	4	5
1. RickettsiabelliiRML369-C		99.1	99.4	98.2	97.8
2. Rickettsiaconoriistr.Malish7	37.2		99.3	98.4	98
3. RickettsiafelisURRWXCal2	39.2	11.6		98.3	97.9
4. Rickettsiaprowazekiistr.MadridE	42.8	58.5	65.0		98.8
5. Rickettsiatyphistr.Wilmington	50.4	66.6	74.0	10.2	

	1	2	3	4	5	6	7	8	9	10
1. ChlamydiamuridarumNigg		98.4	98.6	95.8	95.9	95.7	94.5	94.4	94.4	94.5
2. ChlamydiatrachomatisA/HAR13	25.2		99.9	95.4	95.5	95.1	94	94	93.9	94
3. ChlamydiatrachomatisD/UW3/CX	25.2	0.5		95.5	95.7	95.3	94.2	94.1	94	94.2
4. ChlamydophilaabortusS26/3	40.7	40.5	40.8		99.5	98.4	95.5	95.5	95.4	95.5
5. ChlamydophilacaviaeGPIC	29.0	27.7	27.9	19.7		98.8	95.5	95.4	95.3	95.5
6. ChlamydophilafelisFe/C56	24.0	32.4	32.7	22.2	16.9		95.1	95.1	95	95.1
7. CpneumoniaeAR39	27.8	18.4	18.8	38.0	28.9	35.5		99.9	99.9	100
8. CpneumoniaeCWL029	27.8	18.4	18.8	38.0	28.9	35.5	0.1		99.8	99.9
9. CpneumoniaeJ138	27.8	18.3	18.7	38.0	28.7	35.5	0.2	0.2		99.9

	1	2	3	4	5	6	7	8	9
10. CpneumoniaeTW183	27.8	18.4	18.8	37.9	28.8	35.4	0.2	0.2	0.3

	1	2	3	4	5
1. LactobacillusacidophilusNCFM		92	86.1	86.7	86.1
2. LactobacillusjohnsoniiNCC533	34.5		89.6	89	88.1
3. LactobacillusplantarumWCFS1	83.4	111.3		92.9	90.6
4. Lactobacillussakeisubsp.sakei23K	75.5	98.1	33.0		90.6
5. Lactobacillussalivariussubsp.salivariusUCC118	41.3	33.3	105.1	97.6	

	1	2	3	4	5	6	7	8	9	10	11	12	13
1. Mycoplasmacapricolumsubsp.capricolumATCC27343		79.2	77.7	79	79	79	81.6	99.3	75.6	77.7	79.9	80.1	75
2. MycoplasmagenitaliumG37	80.4		89.7	73.5	73.5	73.5	76.2	78.8	83.1	89.7	76.3	77.5	82.6
3. Mycoplasmahyopneumoniae232	81.3	109.1		72.6	72.7	72.6	76.2	77.5	82.2	98.1	75.7	75.9	81.2
4. Mycoplasmahyopneumoniae7448	177.9	170.1	185.6		99.8	99.8	86.7	78.7	70	72.4	85.8	85.7	70.5
5. MycoplasmahyopneumoniaeJ	175.8	167.8	183.6	3.5		99.9	86.7	78.7	70	72.4	85.8	85.6	70.5
6. MycoplasmahyopneumoniaeJ	174.9	166.6	182.8	4.1	1.3		86.7	78.7	70	72.3	85.9	85.7	70.7
7. Mycoplasmamobile163K	77.2	121.3	79.3	127.9	125.7	124.4		81.2	74	75.9	86.8	85.9	73.4
8. Mycoplasmamycoidessubsp.mycoidesSCstr.PG1	18.1	67.2	71.0	181.0	178.8	177.5	74.5		75.4	77.4	79.9	79.8	74.9
9. MycoplasmapenetransHF-2	45.4	84.8	78.3	210.7	208.5	207.2	99.0	41.1		82.2	72.8	74	86.9
10. MycoplasmapneumoniaeM129	148.5	107.7	87.6	153.8	151.8	151.0	148.0	142.4	153.9		75.4	75.2	81.6
11. MycoplasmapulmonisUABCTIP	56.7	127.6	96.4	156.3	154.2	153.1	44.7	67.4	87.8	161.3		87.8	72
12. Mycoplasmasynoviae53	106.1	94.4	137.8	134.8	132.5	132.6	118.5	113.6	147.8	142.6	109.6		73.3
13. Ureaplasmaparvumserovar3str.ATCC700970	105.0	103.3	117.2	137.4	134.5	134.4	127.6	102.7	117.8	121.4	149.2	118.3	

	1	2	3	4	5	6	7	8	9	10	11	12	13
1. StaphylococcusaureusRF122		99.9	99.9	99.9	99.9	99.9	99.8	99.8	99.9	98.5	98.6	98.4	97.8
2. Staphylococcusaureussubsp.aureusCOL	2.8		100	100	100	99.9	99.9	99.9	100	98.5	98.7	98.5	97.8
3. Staphylococcusaureussubsp.aureusMRSA252	2.9	1.6		100	100	99.9	99.9	99.9	100	98.5	98.7	98.5	97.8
4. Staphylococcusaureussubsp.aureusMSSA476	2.3	1.1	1.1		100	99.9	99.9	99.9	100	98.5	98.7	98.5	97.8
5. Staphylococcusaureussubsp.aureusMW2	2.6	0.9	1.4	0.4		99.9	99.9	99.9	100	98.5	98.7	98.5	97.8
6. Staphylococcusaureussubsp.aureusMu50	3.0	2.0	1.2	1.4	1.6		99.8	99.8	99.9	98.5	98.6	98.4	97.8
7. Staphylococcusaureussubsp.aureusN315	2.7	1.4	1.3	0.8	0.9	1.1		99.7	99.9	98.5	98.7	98.3	97.7
8. Staphylococcusaureussubsp.aureusNCTC8325	2.6	1.0	1.2	0.8	0.8	1.2	1.0		99.9	98.4	98.6	98.3	97.7

9. Staphylococcusaureussubsp.aureusUSA300	3.0	1.2	0.8	1.2	1.1	1.4	1.2	1.0		98.5	98.7	98.5	97.8
10. StaphylococcusepidermidisATCC12228	41.9	41.7	40.5	41.4	41.5	40.5	40.9	41.4	40.6		99.8	98.5	97.8
11. StaphylococcusepidermidisRP62A	43.9	43.7	42.5	43.4	43.5	42.5	42.9	43.4	42.6	3.0		98.6	97.7
12. StaphylococcushaemolyticusJCSC1435	30.7	30.5	29.4	30.2	30.3	29.3	29.6	30.2	29.6	13.8	14.8		98.7
13. StaphylococcussaprophyticusATCC15305	12.3	13.0	12.3	12.6	12.7	12.4	12.3	12.8	97.9	32.0	33.9	26.5	

	1	2	3	4	5	6	7	8	9	10	11	12	13	14	15
1. Streptococcusagalactiae2603V/R		99.9	99.9	93.2	95	95.2	97.1	97	97.1	97.1	97.1	97	97.1	93.7	93.7
2. StreptococcusagalactiaeA909	1.3		99.9	93.1	95.1	95.1	97.2	97.1	97.2	97.2	97.2	97.1	97.2	93.8	93.8
3. StreptococcusagalactiaeNEM316	3.4	3.9		93.1	94.9	95.1	97	96.9	97	97	97	96.9	97	93.6	93.6
5. StreptococcusmutansUA159	37.1	37.2	36.7		93.7	93.8	93.3	93.2	93.3	93.3	93.3	93.3	93.3	94.4	94.4
4. StreptococcuspneumoniaeR6	39.2	40.0	36.5	44.9		99.8	94.8	94.7	94.8	94.8	94.8	94.7	94.8	95.1	95.1
6. StreptococcuspneumoniaeTIGR4	39.1	40.5	36.7	45.5	2.3		94.6	94.5	94.6	94.6	94.6	94.5	94.6	94.9	94.9
7. StreptococcuspyogenesM1GAS	23.2	23.7	22.7	24.1	37.0	36.9		99.9	100	100	100	99.9	100	95.1	95.1
9. StreptococcuspyogenesMGAS10394	22.5	23.1	21.4	25.0	36.2	36.0	1.3		99.9	99.9	99.9	99.9	99.9	95.2	95.2
12. StreptococcuspyogenesMGAS315	23.3	23.8	22.8	25.4	38.9	37.8	2.7	2.9		100	100	99.9	100	95.1	95.1
13. StreptococcuspyogenesMGAS5005	22.8	23.4	22.5	23.5	36.7	36.6	0.7	1.5	2.7		100	99.9	100	95.1	95.1
14. StreptococcuspyogenesMGAS6180	22.3	22.4	21.0	25.4	35.6	36.3	2.3	1.4	3.8	2.5		99.9	100	95.1	95.1
15. StreptococcuspyogenesMGAS8232	23.2	23.7	22.6	24.7	37.8	36.8	2.0	2.2	1.1	1.8	3.2		99.9	95.1	95.1
17. StreptococcuspyogenesSSI-1	23.7	24.3	23.3	25.0	39.0	37.9	2.5	3.0	0.4	2.5	4.0	1.2		95.1	95.1
18. StreptococcusthermophilusCNRZ1066	25.4	26.6	24.2	46.3	22.0	21.8	31.0	29.9	31.0	30.8	30.4	30.7	31.4		100
19. StreptococcusthermophilusLMG18311	25.7	26.8	24.5	47.0	22.5	21.9	31.7	30.5	31.3	31.5	31.0	31.0	31.6	0.9	

	1	2	3	4	5	6	7	8	9	10	11	12	13	14	15	16
1. EscherichiacoliCFT073		99.4	99.4	99.4	98.8	99.4	96.6	97	97.5	97.5	97.5	98.6	98.9	98.8	98.6	99.5
2. EscherichiacoliK12	7.7		99.9	99.9	99	100	96.8	97.2	97.7	97.7	97.6	98.6	99.1	98.9	98.8	99.6
3. EscherichiacoliO157:H7EDL933	3.3	6.6		100	99	99.9	96.8	97.2	97.7	97.7	97.6	98.6	99.1	98.9	98.8	99.6
4. EscherichiacoliO157:H7str.Sakai	3.2	7.1	0.6		99	99.9	96.8	97.2	97.7	97.7	97.6	98.6	99.1	98.9	98.8	99.6
5. EscherichiacoliUTI89	2.8	4.9	4.0	4.2		99	97.4	97.8	97.1	97.1	97.1	99.2	99	99.5	99.4	99
6. EscherichiacoliW3110DNA	7.6	0.3	6.4	6.9	4.7		96.8	97.2	97.7	97.7	97.6	98.6	99.1	98.9	98.8	99.6
7. Salmonellaentericasubsp.entericas...	42.5	35.2	41.5	41.9	40.1	35.4		99.6	99	99	99	97.4	96.7	97.3	97.6	96.8
8. Salmonellaentericasubsp.entericas(2)	44.1	36.7	43.1	43.5	41.6	36.9	1.6		99.3	99.3	99.2	97.8	97.1	97.6	98	97.2
9. Salmonellaentericasubsp.entericas(3)	40.4	33.5	39.4	39.7	37.9	33.7	2.2	3.7		100	99.7	97.1	97.2	97	97.3	97.7

10. Salmonellaentericasubsp.entericas(4)	39.8	32.9	38.8	39.2	37.4	33.1	2.7	4.3	0.6		99.7	97.1	97.2	97	97.3	97.7
11. SalmonellatyphimuriumLT2	43.3	35.9	42.3	42.6	40.8	36.1	1.5	1.3	2.9	3.4		97.1	97.1	97.1	97.3	97.8
12. ShigellaboydiiSb227	6.5	10.5	8.7	8.8	6.8	10.3	45.3	46.8	44.0	43.4	46.4		98.6	99.3	99.5	98.9
13. ShigelladysenteriaeSd197	7.0	10.1	8.8	9.1	6.4	9.9	44.8	46.3	43.6	42.9	46.0	2.6		98.8	98.8	99.1
14. Shigellaflexneri2astr.2457T	5.7	8.8	7.1	7.2	5.3	8.6	43.6	45.1	42.3	41.7	44.7	2.1	3.2		99.5	99.2
15. Shigellaflexneri2astr.301	5.8	9.2	7.4	7.5	5.6	9.0	43.9	45.4	42.7	42.1	45.1	1.9	3.0	0.4		99
16. ShigellasonneiSs046	5.1	9.6	7.5	7.5	5.8	9.3	44.3	45.8	43.0	42.4	45.5	2.0	3.0	1.1	1.2	

	1	2	3	4	5	6	7
1. Bacillusanthracisstr.'AmesAncestor'		100	100	99.9	99.8	99.9	99.9
2. Bacillusanthracisstr.Ames	0.0		100	99.9	99.8	99.9	99.9
3. Bacillusanthracisstr.Sterne	0.1	0.1		99.9	99.8	99.9	99.9
4. BacilluscereusATCC10987	2.3	2.3	2.2		99.7	99.8	99.9
5. BacilluscereusATCC14579	4.9	4.9	4.8	4.6		99.8	99.9
6. BacilluscereusE33L	2.3	2.3	2.2	1.6	3.6		99.9
7. Bacillusthuringiensisserovarkonkukianstr.97-27	1.5	1.5	1.5	1.7	4.8	1.5	
