# Supplementary material for: The reach of the genome signature in prokaryotes
Source: BMC Evol Biol. 2006 Oct 13;6:84. doi: 10.1186/1471-2148-6-84 (PMC1621082; doi:10.1186/1471-2148-6-84)
Supplement: Additional File 4 — Prokaryotic intragenomic genome signature comparisons for species that carry more than 1 chromosome. The numbers correspond to the comparisons depicted in Figure 4. [file 1471-2148-6-84-S4.pdf]

Additional File 4

Prokaryotic intragenomic genome signature comparisons for species that carry more than 1 chromosome. The numbers correspond to the comparisons depicted in Figure 4.

	Number		Accession numbers	Size (bp)
**Alphaproteobacteria**				
	**1**	Agrobacterium_tumefaciens_str_C58_chromosome_circular	NC_003062	2841581
		Agrobacterium_tumefaciens_str_C58_chromosome_linear	NC_003305	2075560
**Alphaproteobacteria**				
	**2**	Brucella_abortus_biovar_1_str_9-941_chromosome_I	NC_006932	2124241
		Brucella_abortus_biovar_1_str_9-941_chromosome_II	NC_006933	1162204
	**3**	Brucella_melitensis_16M_chromosome_I	NC_003317	2117144
		Brucella_melitensis_16M_chromosome_II	NC_003318	1177787
	**4**	Brucella_melitensis_biovar_Abortus_2308_chromosome_I	NC_007618	2121359
		Brucella_melitensis_biovar_Abortus_2308_chromosome_II	NC_007624	1156948
	**5**	Brucella_suis_1330_chromosome_I	NC_004310	2107794
		Brucella_suis_1330_chromosome_II	NC_004311	1207381
**Betaproteobacteria**				
	**6**	Burkholderia_mallei_ATCC_23344_chromosome_1	NC_006348	3510148
		Burkholderia_mallei_ATCC_23344_chromosome_2	NC_006349	2325379
	**7**	Burkholderia_pseudomallei_1710b_chromosome_I	NC_007434	4126292
		Burkholderia_pseudomallei_1710b_chromosome_II	NC_007435	3181762
	**8**	Burkholderia_pseudomallei_K96243_chromosome_1	NC_006350	4074542
		Burkholderia_pseudomallei_K96243_chromosome_2	NC_006351	3173005
	**9**	Burkholderia_sp_383_chromosome_1	NC_007510	3694126
		Burkholderia_sp_383_chromosome_2	NC_007511	3587082
	**10***	Burkholderia_sp_383_chromosome_3	NC_007509	1395069
	**11**	Burkholderia_thailandensis_E264_chromosome_I	NC_007651	3809201
		Burkholderia_thailandensis_E264_chromosome_II	NC_007650	2914771

	**12**	Burkholderia_xenovorans_LB400_chromosome_1	NC_007951	4895836
		Burkholderia_xenovorans_LB400_chromosome_2	NC_007952	3363523
	**13***	Burkholderia_xenovorans_LB400_chromosome_3	NC_007953	1471779
	**14**	Ralstonia_eutropha_JMP134_chromosome_1	NC_007347	3806533
		Ralstonia_eutropha_JMP134_chromosome_2	NC_007348	2726152
**Deinococcus-Thermus**				
	**15**	Deinococcus_radiodurans_R1_chromosome_1	NC_001263	2648638
		Deinococcus_radiodurans_R1_chromosome_2	NC_001264	412348
**Gammaproteobacteria**				
	**16**	Vibrio_cholerae_O1_biovar_eltor_str_N16961_chromosome_I	NC_002505	2961149
		Vibrio_cholerae_O1_biovar_eltor_str_N16961_chromosome_II	NC_002506	1072315
	**17**	Vibrio_fischeri_ES114_chromosome_I	NC_006840	2906179
		Vibrio_fischeri_ES114_chromosome_II	NC_006841	1332022
	**18**	Vibrio_parahaemolyticus_RIMD_2210633_chromosome_I	NC_004603	3288558
		Vibrio_parahaemolyticus_RIMD_2210633_chromosome_II	NC_004605	1877212
	**19**	Vibrio_vulnificus_CMCP6_chromosome_I,_complete	NC_004459	3281945
		Vibrio_vulnificus_CMCP6_chromosome_II	NC_004460	1844853
	**20**	Vibrio_vulnificus_YJ016_chromosome_I,_complete	NC_005139	3354505
		Vibrio_vulnificus_YJ016_chromosome_II,_complete	NC_005140	1857073
	**21**	Leptospira_interrogans_serovar_Copenhageni_str_Fiocruz_L1-130_chromosome_I	NC_005823	4277185
		Leptospira_interrogans_serovar_Copenhageni_str_Fiocruz_L1-130_chromosome_II	NC_005824	350181
	**22**	Leptospira_interrogans_serovar_Lai_str_56601_chromosome_I	NC_004342	4332241
		Leptospira_interrogans_serovar_Lai_str_56601_chromosome_II	NC_004343	358943
		**Total number of chromosome sequenecs**	**42**	

***) For species with three chromosomes, we only analysed the δ* values between chromosome 1 and chromosome 2, and between chromosome 1 and chromosome 3.**
